# Supplementary figures and images for: Alk‐Fam150b (augmentor α) expression in the paraventricular nucleus of the mouse hypothalamus at molecular resolution, and its sensitivity to acute stress
Source: J Neuroendocrinol. 2026 Mar 19;38(3):e70159. doi: 10.1111/jne.70159 (PMC13002349; doi:10.1111/jne.70159)

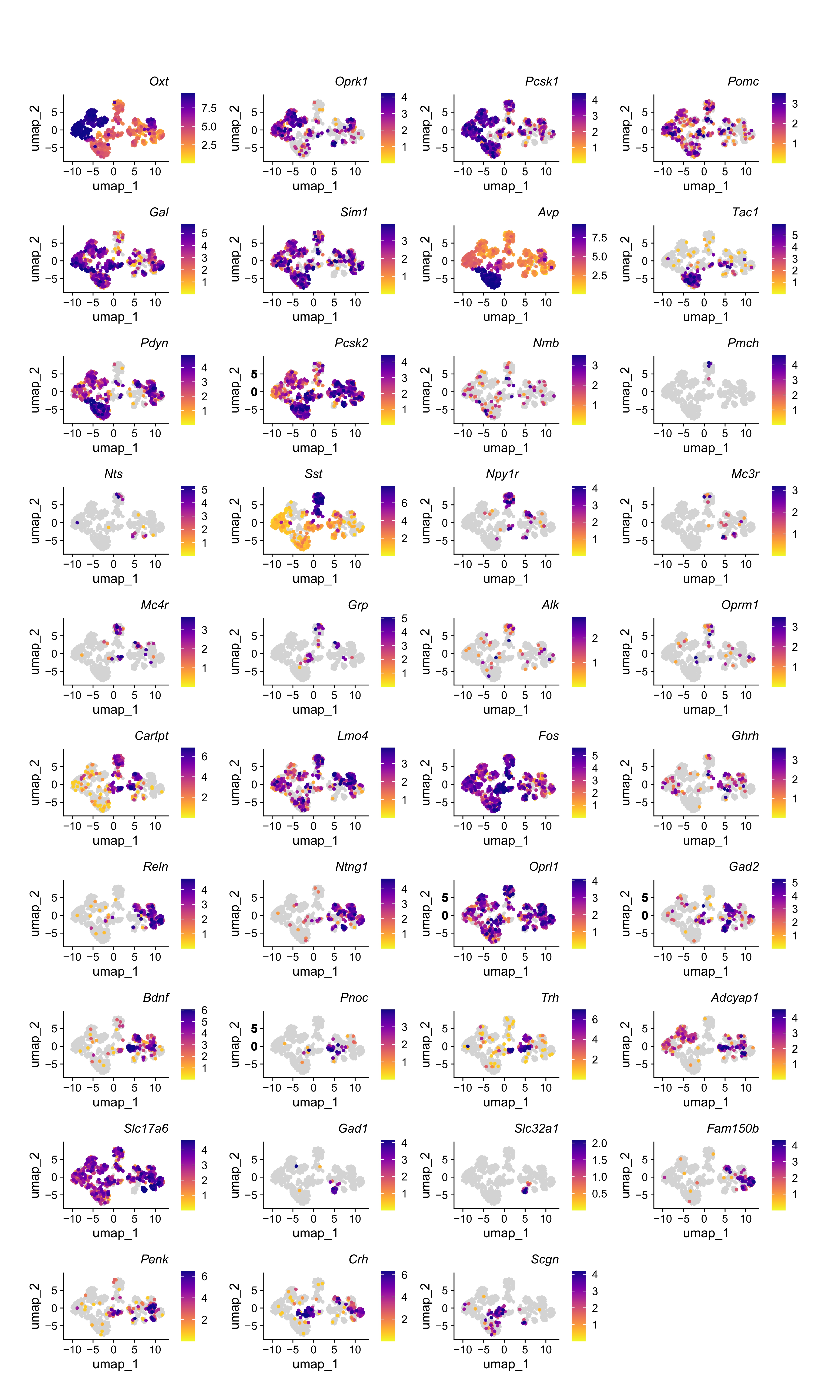

Supplement: Supplementary file 1 — FIGURE S1. Expression landscape of common neuropeptides and receptors in the mouse PVN. UMAP plots depict normalized expression levels for selected genes in all neurons retrieved from a reference single‐cell RNA‐seq dataset (Smart‐seq2). 25 [file JNE-38-e70159-s003.jpg]

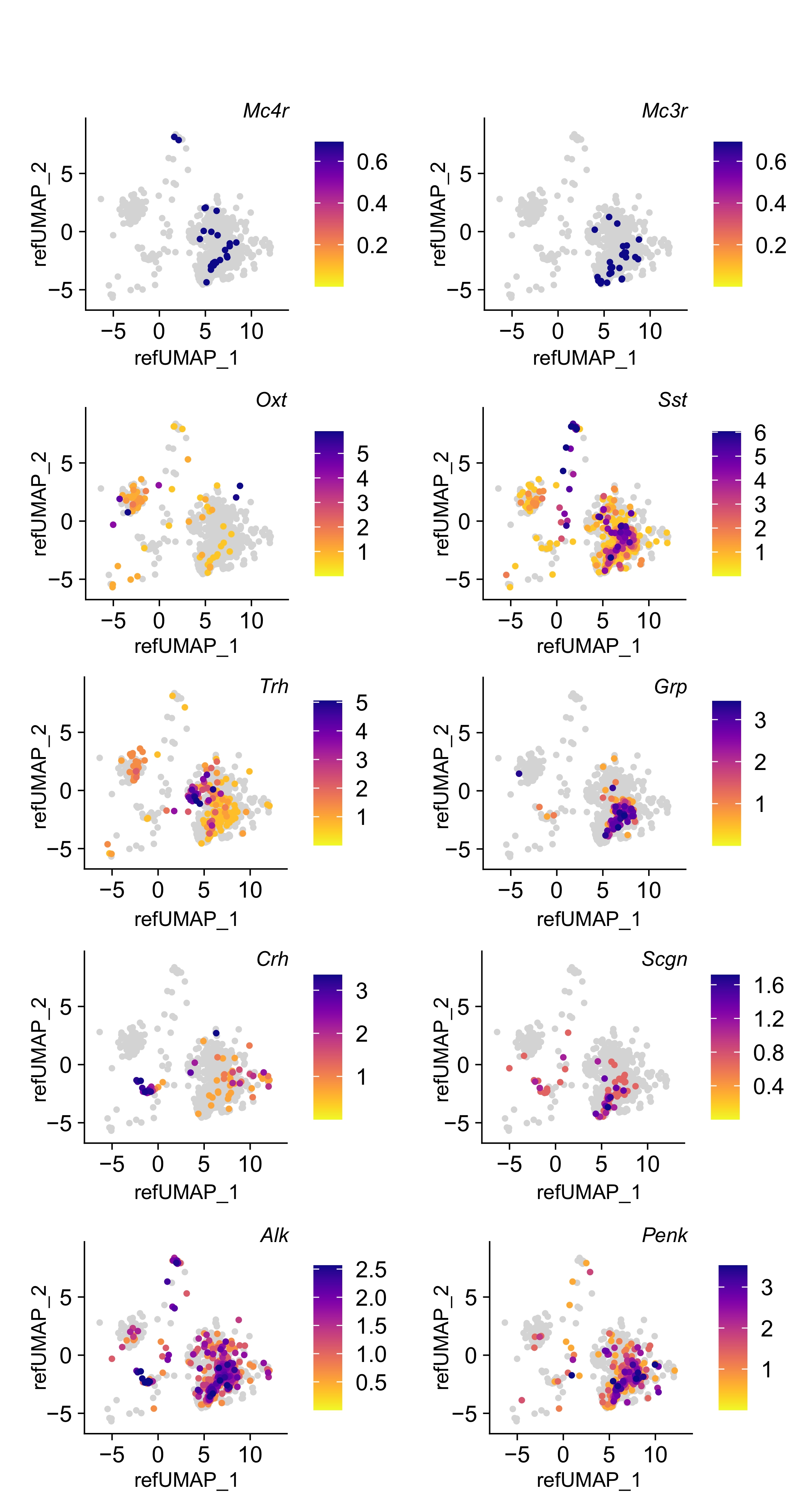

Supplement: Supplementary file 2 — FIGURE S2. Focused expression of marker and regulatory genes. UMAP plots for the expression of Mc4r, Mc3r, Oxt, Sst, Trh, Grp, Crh, Scgn, Alk, and Penk from 10× data 42 mapped onto a reference UMAP from Xu et al. 25 [file JNE-38-e70159-s005.jpg]

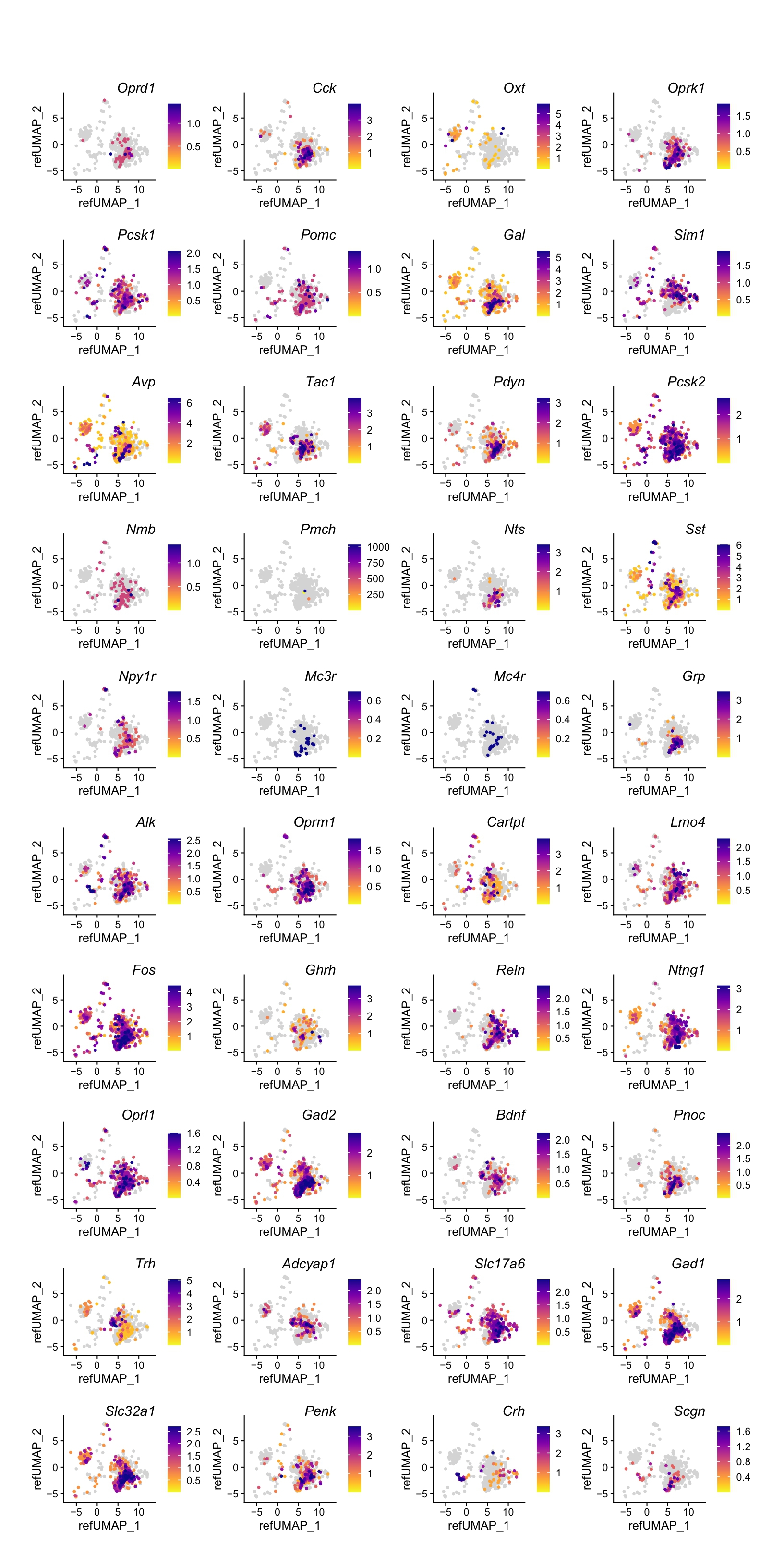

Supplement: Supplementary file 3 — FIGURE S3. Expression landscape of neuropeptides and neuropeptide receptors mapped onto an integrated Smart‐seq2 reference. UMAP plots show the expression of genes selected from 10× data 42 and mapped onto an integrated reference UMAP from Xu et al. 25 [file JNE-38-e70159-s001.jpg]

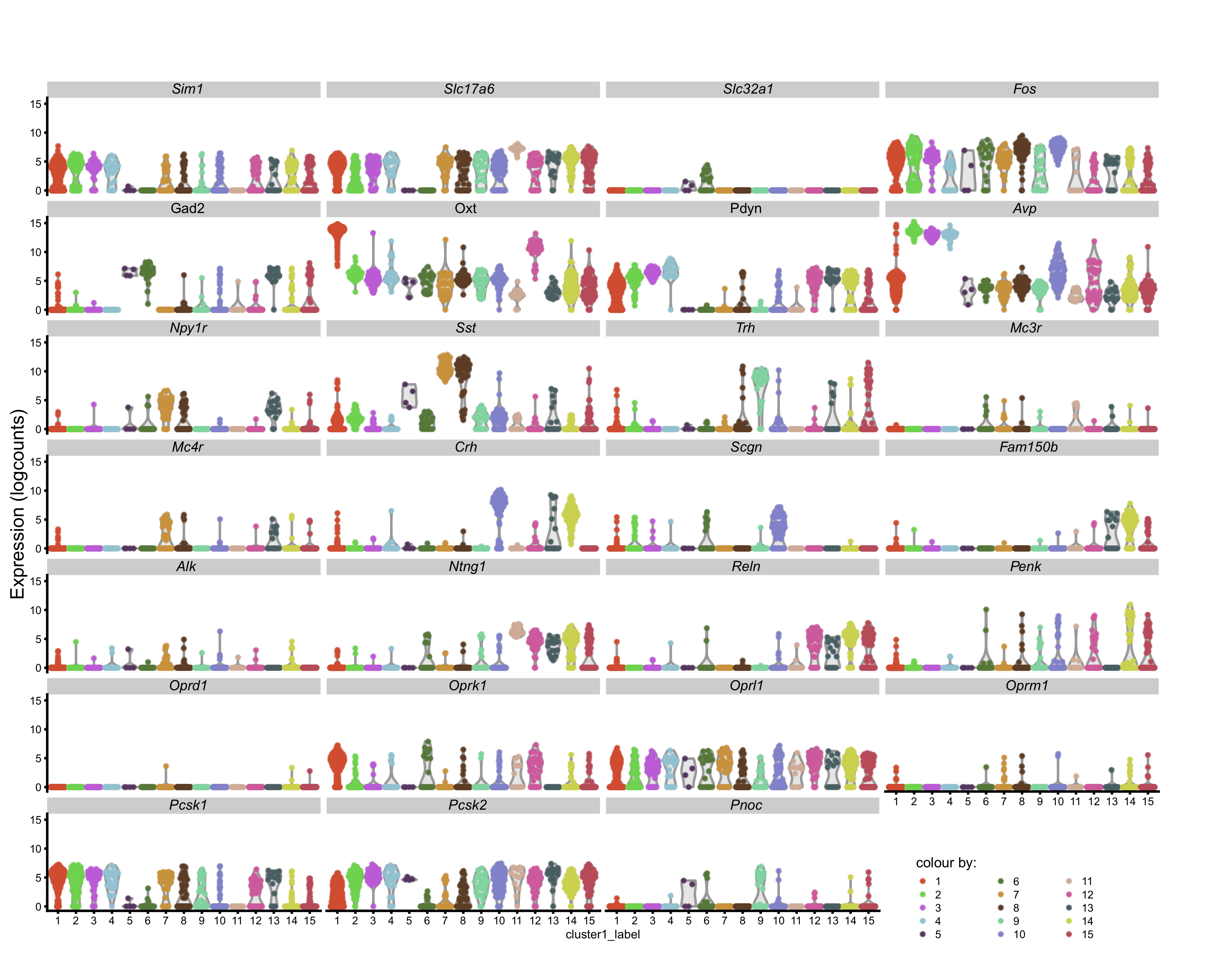

Supplement: Supplementary file 4 — FIGURE S4. Quantification of gene expression across neuronal clusters in the PVN. Violin plots illustrate the distribution of normalized expression levels (logcounts) for selected genes across cell clusters (not sorted) identified in the PVN by Smart‐seq2. 25 Each violin plot shows the density distribution of expression for a specific gene within a given cluster, providing a quantitative comparison of gene expression profiles across neuronal subpopulations. [file JNE-38-e70159-s002.jpg]

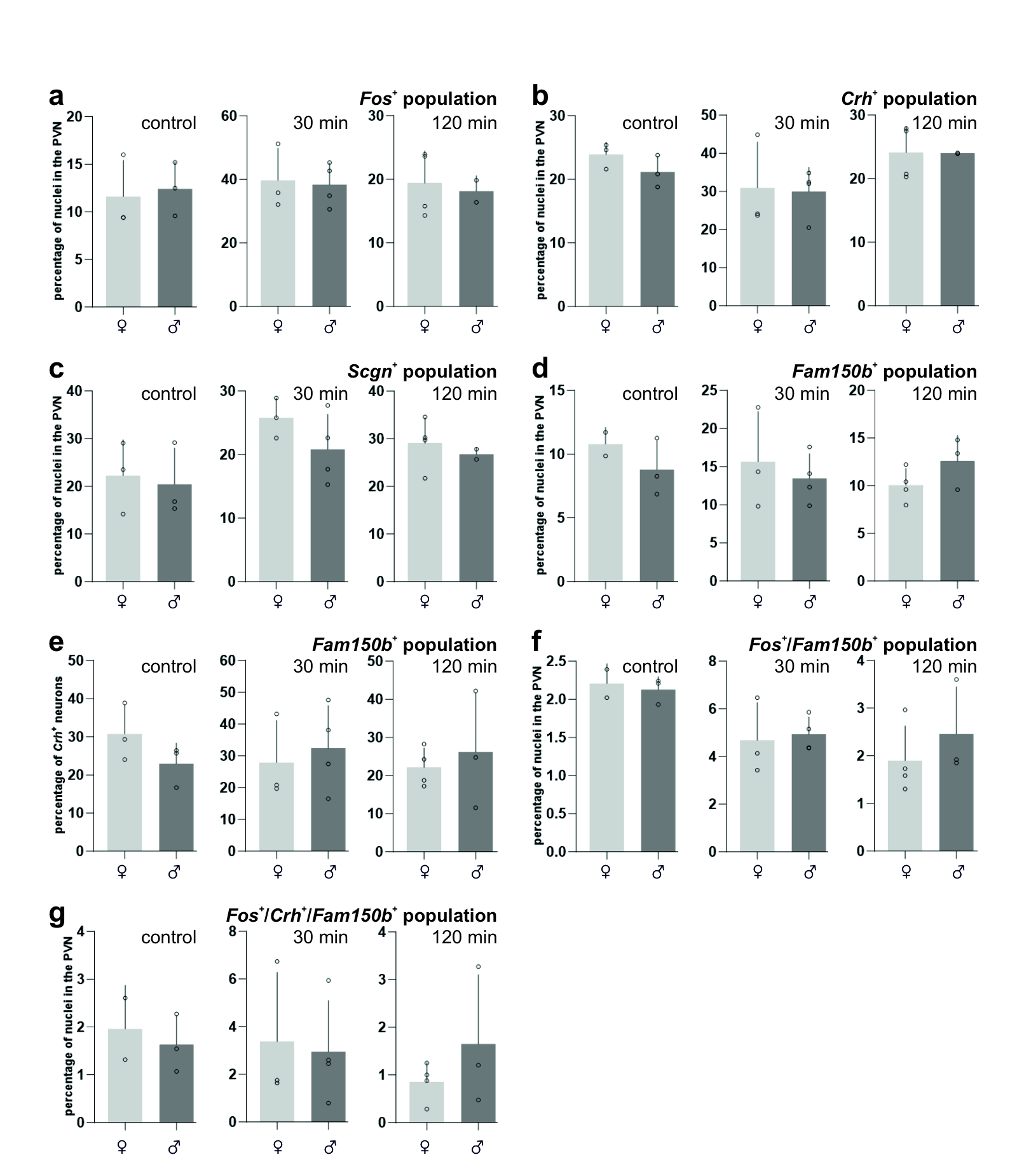

Supplement: Supplementary file 5 — FIGURE S5. Effects of sex on mRNA expression in the PVN. Comparison of the number of cells expressing Fos (a), Crh (b), Scgn (c) or Fam150b (d) in the PVN between female and male mice in three experimental conditions (control, ‘30 min’, and ‘2 h’ after stress induction). Results were normalised for the entire PVN. (e) Sex effects of the number of Crh + neurons expressing Fam150b in the PVN under experimental conditions. (f) Comparison of cell numbers co‐expressing Fos/Fam150b within the PVN in females vs. males. (g) Cell numbers for Fos +/Crh +/Fam150b + neurons in the PVN of female and male experimental subjects. A p value of <0.05 was considered statistically significant. [file JNE-38-e70159-s006.jpg]

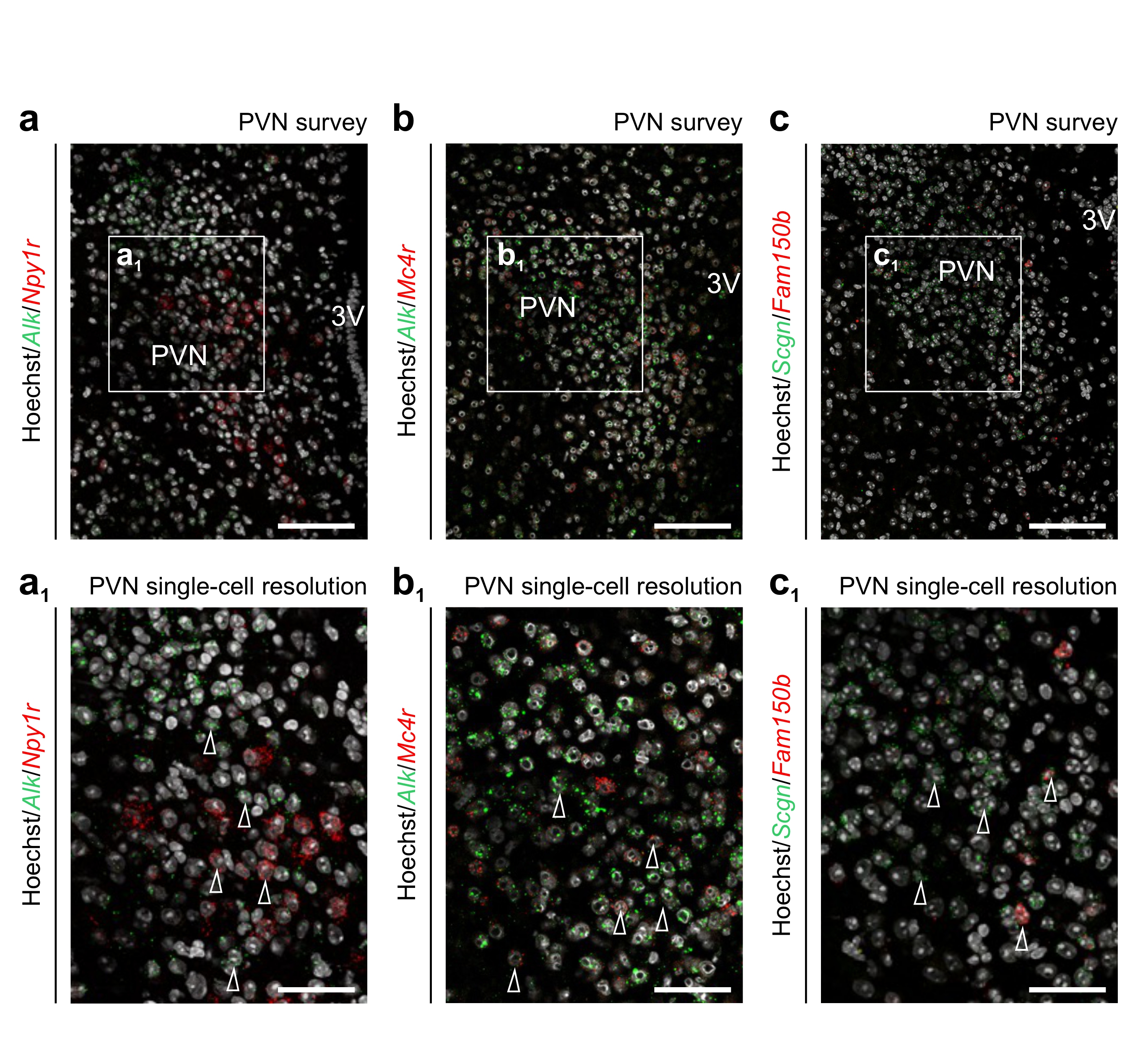

Supplement: Supplementary file 6 — FIGURE S6. Colocalization of Alk and Fam150b with other cellular markers in the PVN. Multiple labelling in situ hybridization for Alk and either Npy1r (a) or Mc4r (b) in the PVN. Alk did not seem to co‐localize with either Npy1r or Mc4r, two receptors implicated in the control of metabolism and feeding behaviors. 20 , 21 , 22 , 23 , 24 (c) Multiple labelling in situ hybridization for Fam150b and Scgn in the PVN. Fam150b and Scgn did not co‐localize in the PVN, confirming earlier data by single‐cell RNA‐seq. 25 Open rectangles show the positions of high‐resolution insets (a1, b1, c1), which illustrate mRNA distribution at cellular resolution. Scale bars = 300 μm (overviews) and 30 μm (insets). [file JNE-38-e70159-s004.jpg]
